# Time-of-Flight Secondary Ion Mass Spectrometry (ToF-SIMS): A New Tool for the Analysis of Toxicological Effects on Single Cell Level

**DOI:** 10.3390/toxics4010005

**Published:** 2016-02-15

**Authors:** Harald Jungnickel, Peter Laux, Andreas Luch

**Affiliations:** Department of Chemicals and Product Safety, German Federal Institute for Risk Assessment, Max-Dohrn-Strasse 8-10, 10589 Berlin, Germany; harald.jungnickel@bfr.bund.de (H.J.); andreas.luch@bfr.bund.de (A.L.)

**Keywords:** ToF-SIMS, imaging mass spectrometry, MIMS, lipidomics, cryopreservation, single cell analysis

## Abstract

Single cell imaging mass spectrometry opens up a complete new perspective for strategies in toxicological risk assessment and drug discovery. In particular, time-of-flight secondary ion mass spectrometry (ToF-SIMS) with its high spatial and depth resolution is becoming part of the imaging mass spectrometry toolbox used for single cell analysis. Recent instrumentation advancements in combination with newly developed cluster ion guns allow 3-dimensional reconstruction of single cells together with a spatially resolved compound location and quantification on nanoscale depth level. The exact location and quantification of a single compound or even of a set of compounds is no longer restricted to the two dimensional space within single cells, but is available for voxels, a cube-sized 3-dimensional space, rather than pixels. The information gathered from one voxel is further analysed using multivariate statistical methodology like maximum autocorrelation factors to co-locate the compounds of interest within intracellular organelles like nucleus, mitochondria or golgi apparatus. Furthermore, the cell membrane may be resolved, including adhering compounds and potential changes of the lipid patterns. The generated information can be used further for a first evaluation of intracellular target specifity of new drug candidates or for the toxicological risk assessment of environmental chemicals and their intracellular metabolites. Additionally, single cell lipidomics and metabolomics enable for the first time an in-depth understanding of the activation or inhibition of cellular biosynthesis and signalling pathways.

## 1. Introduction

Time-of-flight secondary ion mass spectrometry (ToF-SIMS) in combination with stable isotope labelling and a range of new cluster ion beams for depth profiling has established the opportunity for exact identification and quantification of substances on a cellular and subcellular level. This allows for the first time single cell analysis in combination with a full 3-dimensional reconstruction to assess the metabolite distribution pattern. Single cell imaging can also mirror the intracellular distribution of cell specific biomolecules. These may be synthesised either by the target cells themselves or in specific tissues or organs from where they are subsequently transported to their respective destinations. Subsequently to their uptake, the biomolecules may either be stored in cell-specific organelles or further metabolised. Reaction products are used either specifically at their site of origin or transported out of the cell again and delivered to other targets. This is achieved by a complex framework of cellular intake, uptake and segregation which can be observed not only in tissues but also in single cells. Different single cells in a tissue or organ may represent either different metabolic states or may participate in different metabolic frameworks. This may include a specific up- or down-regulation of toxicological relevant signalling pathways. Even in complex tissues single cell analysis is of paramount interest in toxicological research, especially with respect to the identification of the early onset biomarkers of diseases such as cancer. The gathered information can also be used for drug discovery and is further instrumental in the assessment and prediction of possible drug and its metabolites’ distributions as well as of the fate and metabolism of environmental chemicals and their associated toxicity.

## 2. Cryopreservation of Cells in a Native State

Conservation of the native status and distribution of all biomolecules is the most important prerequisite for single cell analysis. To achieve this, a plunge freezing methodology is used commonly [1], where the sample is plunged with high speed into a cryogen, in most cases liquefied isopentane or propane. By the application of this procedure the formation of large ice crystals, which could cause the dislocation of small molecules or even rupture of intracellular membranes or the cell membrane is avoided. Equally important is the introduction of a washing step prior to cryopreservation in order to remove excess salt concentrations from the sample surface, which may influence the imaging analysis significantly. Normally either ammonium acetate [2] or ammonium formate [3,4] are used at physiological concentrations, which avoids cell rupture by the washing solution. The application of this methodology enables co-localisation of substances in the cell nucleus, the intracellular space or at the cell membrane, allowing for the exact location of metabolites and externally added compounds like environmental chemicals or drugs. One important advantage of the technology is that the target compound is imaged label-free and thus without the use of additional marker substances. The application of a specific fast-freezing plunger technology, which conserves the native state of the cells without creating artefacts like chemical modifications or ice crystals, allows for an exact substance localisation, picturing the real-life state of the cell. Cryopreservation in combination with enhanced 3-dimensional imaging capacity, enabled by newly developed cluster ion beams for ToF-SIMS, could also be used to distinguish single cells *in vitro* or in tissue sections according to their physiological state.

## 3. Progress of ToF-SIMS Instrumentation: New Ion Cluster Guns and High Mass Resolution

In recent bioanalytical ToF-SIMS applications, cluster ion guns allow for a deposition of incident energy close to the sample surface and help for an effective detachment of organic molecules as intact ions with limited surface damage [5,6,7]. The development of new cluster ion guns prepares the ground for the sub-micrometer lateral localisation of biomolecules which was shown to be in principle achievable for example by the bismuth (68 nm, [8], the C_60_^+^ (less than 200 nm, [9]) and the gold (*ca.* 400 nm, [10]) cluster ion guns. The surface sensitivity of primary ions is linked to the depths of the impact crater, primary ion implementation and molecular ion escape. A study performed with different bismuth and C_60_ primary ions on trehalose and tetraglyme films has suggested a large impact of surface erosion by the primary ion source, a parameter also affected by sample characteristics such as density [11]. Currently, the applications of ToF-SIMS onto biological samples are still limited and require further adaptation work until a 3-dimensional reconstruction of whole cells based on mass spectral data can be achieved. The capability for identification of biomolecules is provided by the coupling of new ToF-SIMS methodology to tandem mass spectrometry in combination with high mass resolution. There are currently two methods available which deliver promising results for depth profiles of biological matrices and single cells. One is the recently developed argon gas cluster ion beam, which can be used in a dual beam mode with a bismuth cluster ion gun. The new buncher technology enables the decoupling of the sputtering process from the mass spectral analysis, allowing simultaneously for high spatial and mass resolution. In order to achieve this, secondary ions are sampled in a shape field buncher and directed further from there into the ToF-SIMS by a reflectron. Integration of a collision cell in between of buncher and reflectron allows for analysis in tandem mass spectrometry or common time-of-flight mode [12]. A major advantage of this technology is the prevention of material loss during the sputtering event, resulting from an interleaved dual ion beam, where a pulsed ion cluster ion beam is used to acquire an image followed by a continuous polyatomic ion beam to sputter the material. This combination enables for 3-dimensional analysis of biological materials with a depth resolution down to less than 10 nm [13] and simultaneously retains all molecular information from the sample. A second approach uses a C_60_^+^ primary ion source (40 keV) with a nanoscale spot size, where the ion beam can be used in continuous mode simultaneously etching and imaging [14]. The C_60_^+^ ion source may be coupled with a commercially available triple-quadrupole orthogonal time-of-flight mass spectrometer with tandem mass spectrometry [15]. An orbitrap mass spectrometer is combined to SIMS in a current research project led by the National Physical Laboratory, London, UK [16]. Due to high mass resolution and accuracy a sensitivity enhanced by two orders of magnitude and molecular imaging in 3D with 50 nm spatial resolution are intended. For continuous mode analysis of biological samples an argon gas cluster ion beam was developed [13]. Preliminary results for *in vitro* cultured cancer cells and cryo-sectioned tumour samples showed for Ar_1000_^+^ and Ar_2000_^+^ clusters increased signal persistence from phospholipid molecular ions as well as a significant reduction in the chemical background noise in comparison to C_60_^+^ [17]. Angerer *et al.* demonstrated the suitability of 40 keV Ar_4000_^+^ clusters in combination with trifluoric acid treatment to achieve a high resolution, lipid signals from mouse brain samples could be increased 30 to 50 fold [18]. By the use of modern imaging technology for processing of the acquired data the exact localisation of single compounds within a single cell is revealed. The result is an image of the acquired sample where all the chemical information is stored within 3-dimensional voxels rather than two dimensional pixels.

### 3.1. Bacterial Phenotyping

An example for the application of the buncher technology in combination with a C_60_^+^ ion gun comprises the differentiation of the bacterial species *Pseudomonas aeruginosa*, *Escherichia coli* and *Staphylococcus aureus*. Principal component analysis of positive ion mode data revealed m/z 272.2 as a major difference between the two Gram-negative species *Pseudomonas aeruginosa* and *Escherichia coli*. Tandem mass spectrometry identified the fragment ions at m/z 159 and 172 as dissociation products of m/z 272.2 [19]. 4-Hydroxy-2-alkylquinolines produced by *Pseudomonas aeruginosa* are associated to m/z 272 and both dissociation fragments [20]. Hydroxyalkylquinolines represent signaling molecules and are assumed to be accessible to ToF-SIMS analysis due to their location at the outer surface of the cells. 

### 3.2. Imaging of Mammalian Cervical Carcinoma Cells

The buncher technology together with a C_60_^+^ ion gun in a single ion beam continuous mode was successfully used to image mammalian HeLa cells *in vitro* [21]. Three-dimensional pictures of single HeLa cells where generated from acquired mass spectral data. By application of multivariate data analyses, cell nucleus and intracellular space were discriminated; the cell membrane could be successfully distinguished and visualised using phospholipid ions. Specific biomolecules, like the DNA bases adenine and guanine could be attributed to the cell nucleus. Additionally specific depth related pattern changes could be observed for phospholipid membrane lipids and nuclear DNA base molecules. The complementation of ToF-SIMS analysis with atomic force microscopy enabled to calculate the exact height of a single HeLa cell with 520 nm [22]. 

### 3.3. Association of Cholesterol Distribtion and Alzheimer Disease 

Furthermore, ToF-SIMS analysis of human brain revealed a significantly higher cholesterol signal in the lower half of the cerebral cortex of Alzheimer disease patients. Cholesterol overload of this tissue was concluded a modification linked to Alzheimer disease [23]. In a mouse model of Alzheimer disease, an altered cholesterol association near to amyloid deposits was discovered by a ToF-SIMS investigation in combination with fluorescence microscopy [24]. An analysis of amyloid β-peptides, which are considered key molecules for the understanding of Alzheimer disease, confirmed a lower influence of matrix components and a focus on larger secondary ions associated with biomolecules for the use of the argon cluster gun in comparison with the bismuth cluster gun [25]. Further adaptation of ToF-SIMS analysis to small sized mammalian cells is needed. It is representing a perspective for the assessment of biochemical processes in neuronal degradation such as observed in Alzheimer’s and Parkinson’s disease.

### 3.4. Behaviour of Hydroxypropylcellulose during the Granulation of Pharamceuticals

The behaviour of hydroxypropylcellulose (HPC), m/z 59, during the wet granulation process of pharmaceutic tablets and capsules was elucidated with a ToF-SIMS instrument operated with a 30 kV bismuth ion source. Monitoring of the granule surface revealed an increased presence of the compound during wet granulation linked to a decrease of other molecules. The results were confirmed by inverse gas chromatography. As an explanation, dissolution of HPC in the spray water and a squeeze out effect during the granulation process are considered [26]. The application demonstrates the suitability of ToF-SIMS for a detailed analysis of surface molecules.

## 4. Multi Isotope Imaging Mass Spectrometry (MIMS)

Contrastingly to the ion cluster guns in ToF-SIMS that allow direct detachment and analysis of intact surface molecules, these are split into fragments by the energetic ion beam used by a technology originally introduced as scanning secondary ion analytical microscopy with parallel detection [27], today also called nano-SIMS. Either Cs^+^ or O^−^ primary ions are used; the loss of structural information is encountered by labelling with stable isotopes and their subsequent detection by a high resolution magnetic mass analyser. Employment of co-axial optics enables for focussing of the primary ion beam and collection of secondary ions in parallel [7,28]. Up to seven masses are simultaneously imaged and quantified with this setup [29,30]. A spatial resolution down to 50 nm was confirmed in a study on the distribution of triglyceride-rich lipoproteins in heart capillaries and heart capillary endothelial cells of mice [31]. Stable isotopes have now been used in drug discovery for a long time not only to monitor the fate of the drug in the tissue, but also to quantify and assess drug metabolite concentrations in a time-dependent manner. Using stable isotopes in SIMS analyses has opened the new area of multi-isotope imaging mass spectrometry (MIMS) [32,33], a methodology for quantitative imaging of single cellular uptake and subsequent intracellular distribution of biomolecules. This technique enables for localisation of compounds within different cell compartments, but also for investigation of chemical and drug metabolism. For the first time, drugs and their metabolites can be imaged and quantified in single cells, allowing the exact assessment of drugs not only in a time-dependent manner, but also in different single cells. Application of these methods offers the opportunity to design drugs that specifically affect their target cells and to reduce significantly the dose required for treatment success. This may contribute to the reduction of drug-induced side effects.

### 4.1. MIMS and the Immortal Strand Hypothesis

MIMS experiments were used to investigate the “immortal strand hypothesis” [34]. The hypothesis predicts that in asymmetric stem cell division chromosomes with older DNA templates transfer to the daughter cells, which then remain stem cells. Earlier conducted autoradiography studies proposed long-living small intestine crypt cells, which retained the radioactive isotope label over a very long time. MIMS experiments showed for the first time that when using stable isotopically labelled ^15^N-thymidine, equal incorporation rates of the label into daughter cells occur. No long-term stem cells with the label were observed with MIMS in the small intestine crypts. 

Another application of MIMS has investigated mammalian heart renewal [35]. ^15^N labelling of thymidine and its incorporation into DNA revealed for the first time that especially in aging cardiomyocytes, but also after injury, cell division originated from already pre-existing cardiomyocytes rather than from invading stem cells. The resulting cells showed polyploidy and multi-nucleation, but also diploid and mono-nucleate cardiomyocytes were generated in this process.

### 4.2. Incorporation of Trehalose into the Nucleus of Mouse Sperm Cells

As a further discovery, the incorporation of ^18^O-labelled trehalose into the nucleus of single mouse sperm cells was revealed by MIMS [36]. Trehalose is used as a beneficial compound for mouse sperm storage at above freezing point temperatures because it preserves the developmental potential of partially desiccated mouse sperm cells.

### 4.3. Internalisation of p-boronphenylalanine by Human Glioblastoma Cells

ToF-SIMS in combination with stable isotope labelling was used to monitor the time-dependent incorporation and subcellular distribution of *p*-boronphenylalanine, a clinically approved boron neutron capture therapy agent for cancer therapy [37], in the T98G cell line that was derived from a human glioblastoma tumor. The study investigated the incorporation of ^10^B-labelled *p*-boronphenylalanine and the incorporation of ^13^C^15^N-labelled phenylalanine. It was shown that both compounds accumulated at higher doses within single cells at 6 h upon 2 h of exposure. Intracellular accumulation of *p*-boronphenylalanine however, was diminished in the mitochondria-rich perinuclear region in comparison to phenylalanine, indicating different intracellular metabolic processes in mitochondria for phenylalanine and *p*-boronphenylalanine. The study shows clearly that not only the overall uptake of pharmaceuticals is of vital interest in drug discovery, but also the intracellular location of these pharmaceuticals. 

### 4.4. Glutamine Metabolism in Breast Cancer Cells

Glutamine labelled with the isotope ^15^N was measured by MIMS in single MCF-7 breast cancer cells [31]. The compound is the most abundant free amino acid in the human body with significant accumulation in cancer cells, where it is metabolised and converted to proteins and nucleotides. Glutamine is also important for the maintenance of the mitochondrial membrane potential and integrity. Picturing of single cells by nano-SIMS and an energy selective backscattered electron detector revealed accumulation of ^15^N-labelled glutamine not only in the nucleolus and the nucleus, but also in the Golgi apparatus and the endoplasmatic reticulum. Interestingly, the same methodology can be used to estimate the turnover rate of single cells exposed to labelled glutamine for different periods in conventional pulse-chase experiments. 

### 4.5. Uptake of Chemotherapeutics by Breast Cancer Cells

The MIMS methodology is capable to investigate the distribution of drugs and their metabolites simultaneously with cell prone biomolecules within different cell compartments. This may serve the investigation of drug-induced metabolism of cell native compounds and the origin of signalling cascades. One example is the successful imaging of a new group of platinum-based chemotherapeutics, triplatin nc, within single MCF-7 breast cancer cells and in different subcellular structures using the signal from ^195^Pt and ^15^N, respectively [38]. The subcellular distribution of chemotherapeutics enables a deeper understanding of drug metabolism and drug target identification. The study showed that in an early stage, 1 h after drug uptake, there is drug accumulation in distinct areas of single cells, which may be endocytic vesicle-like structures close to the perimeter of the cell. The study further showed that at that early time points, there is no co-localisation of drug molecules with DNA molecules, visualised within single cells using the ^31^P signal. After 2 h the drug accumulated in vesicle like-structures throughout the entire cytoplasm and a clear increase in drug concentration within single cells was observed. Distribution comparison to cisplatin, a well-known chemotherapeutic used in cancer treatment, showed distinct differences in the overall location of the two drug molecules within single cells. In comparison to triplatin nc, cisplatin was not taken up by the cells in a similar amount and did not accumulate in distinct hot spots and vesicle-like structures. However, an even distribution in the whole cell cytoplasm was observed at the end of a 2 h exposure period. Already after 2 h, an accumulation of triplatin nc could be observed within the nucleus and the nucleoli, indicating the interaction of the drug molecules and genomic DNA. Due to the dual label of the drug compound with ^195^Pt and ^15^N, it was possible to show a partial metabolism of the triplatin nc complex already 2 h after drug exposure of MCF-7 cells at some spots, indicated by a high ^195^Pt signal and very low to none ^15^N signal. Therefore the methodology may also be used for identification of drug metabolism in single cells including target cancer cells. 

## 5. New Developments towards ToF-SIMS Quantification: Some Examples

Quantitative measurements with ToF-SIMS are challenging due to different ionisation efficacies and the strong influence of matrix compounds on this parameter [39]. The use of multivariate analysis in combination with ToF-SIMS data is an approach directed to the quantification of organic compounds in complex matrices. Here especially data normalisation and the exact discrimination between substance specific mass peaks and background noise are of paramount importance to achieve exact quantitative data [40]. This approach was already successfully applied for the quantification of organic compounds in mixed organic layers and of cholesterol in supported lipid membranes [41]. Further developments of the method could enable to quantify a multitude of biomolecules in single cell systems. Numerous diseases and biological disorders can be correlated to an imbalance in essential heavy metal ions, which serve either as co-factors for enzymes or essential parts of important biomolecules such as vitamins or haemoglobin. The exact distribution of heavy metals within single cells is therefore of outmost importance not only in the diagnosis of specific disorders but also in the assessment of recovery strategies or drug treatment of heavy metal imbalance-induced diseases.

### 5.1. Quantification of Choline in Motor Neurons

Intracellular choline, a metabolic precursor of the neurotransmitter acetylcholine, was visualised in spinal motor neurons of humans by multivariate interpretation of ToF-SIMS data. Here a new approach developed for imaging mass spectrometry, a maximum autocorrelation factor analysis, showed clearly that a high concentration of choline is located in α-motor neurons [42]. This methodology could be used in the future to assess -motor neuron integrity and activity using the marker compound choline for imaging mass spectrometry.

### 5.2. Calcium Redistribution in Certain Cell Types is Associated with Retinal Ischemia

ToF-SIMS was used to image calcium and enzymes in ischemic rat tissue [43]. The distribution of calcium is of special interest due to its importance in receptor activity, ion channel activation and also as a co-factor for enzymes. Mammalian retina is normally a multi-layered tissue, comprising of three nerve cell layers, that is, the ganglion cell layer, the inner and the outer nuclear cell layer, followed by two layers of synapses, the inner and the outer plexiform layer. Ischemia can affect all cell types and ultimately results in cell death and apoptosis. ToF-SIMS studies could reveal calcium redistribution within different cell types being associated with retinal ischemia. In ischemic retina, calcium accumulation could be observed in the plexiform layers 12 h after ischemia induction, comprising axons and dendrites first, progressing to the inner nuclear layer after 24 h, and finally reaching the outer nuclear layer at 48 h after ischemia induction. The calcium accumulation correlated very well with apoptosis. Therefore, irregular calcium distributions within the retinal tissue or single cells thereof may indicate retinal disorders or even ischemia in its very early state.

### 5.3. Conversation of Strontium from Implants to Extracellular Bone Matrix

Strontium is used for osteoporosis treatment in newly developed therapeutic implants [44]. With the help of ToF-SIMS it was shown for the first time that strontium enrichment not only occurs within single human mesenchymal cells, but also in the mineralised extracellular matrix. This indicates that strontium used for the implant is taken up by human mesenchymal stem cells and converted into the extracellular bone matrix. High strontium intake by mesenchymal stem cells promotes not only cell replication but also osteoblastic cell differentiation thereby reducing apoptosis.

### 5.4. Single Cell Analysis of Aquatic Organisms

For the first time a 3-dimensional reconstruction of a single cell, a *Xenopus laevis* oocyte, based only on acquired mass spectral information processed with multivariate statistics became reality [14]. The outer cell membrane of the oocyte could be visualised in a 3-dimensional reconstruction based on ions from phosphatidyl choline, a cell membrane phospholipid. Single neurons from the sea slug *Aplysia californica* were used for imaging of the vitamin E distribution by the application of a ToF-SIMS C_60_^+^ ion gun in combination with tandem mass spectrometry [15]. It was shown that the compound preferably accumulates in the cell membrane and within the cell membrane at the junction of cell soma and neurites of the neurons compared to other cell subcompartments. While vitamin E and cholesterol coud be detected as intact molecules, other biomolecules were recorded as fragment ions. For the major membrane lipid, 1-hexadecyl-2-octadecenoyl-*sn*-glycero-3-phosphocholine, the biological salt adducts of m/z 768.6 [M+Na]^+^ and 784.6 [M+K]^+^, their high mass ions at m/z 709.5 and 725.5 and the phosphatidylcholine headgroup at m/z 184 were observed. The summarised total contribution of these phosphatidylcholine-derived peaks shows a heterogeneous distribution of the substance on the neuron. As the dominant phospholipid of the neuron surface, phosphatidylcholine is a precursor of the platelet-activating factor which is involved in regulation of inflammation [45]. 

### 5.5. Nickel Distribution in Plants

The localisation of nickel in the accumulator plant *Alyssum lesbiacum* was investigated [46]. An accumulation of the element was observed in subsidiary and epidermal cells, alongside with a uniform distribution in the central vacuoles. Lower nickel concentrations were observed in guard and mesophyll cells, which are metabolically active cell types. Regardless of this observation, a significantly higher amount of nickel was confirmed for cell walls in general. Interestingly the element showed a negative correlation with phosphorous. While high concentrations of nickel and low concentrations of phosphorous occurred in the cell walls and the vacuolar lumen, low concentrations of nickel and high concentrations of phosphorous were found in the metabolically active cytoplasm. Also in the tripartite junctions at the base of plant trichomes, small outgrowths from the epidermis, nickel accumulation could be observed, reflecting the affinity of nickel to the middle lamella and cell wall.

## 6. Lipidomics

Lipidomics comprises the analysis and quantification of cellular lipids to gain information about differences of membrane compositions as well as on the metabolic state of body liquids, tissues and even single cells. The revealed information may be used afterwards not only for the diagnosis of various diseases like cancer, Alzheimer’s disease, and diabetes but also for the assessment of early onset disease biomarkers and drug treatment success. Analysis and comparison of single cells’ lipid profiles provides insight into specific metabolic states in contrast to conventional lipidomics dealing with cell colonies, tissues or even whole organs. 

Within a cell colony or a tissue section specific cells may have their own lipid profiles indicating not only the state of the single cell in respect to its signalling status or metabolic state, but also hinting about hot spot regions, where cells develop individual responses to environmental chemicals like apoptosis or tumour formation. Analysing and characterising lipid profiles from single cells therefore enables sensitive detection of aberrant lipid profiles as markers for early disease development, which might be overlooked due to their small concentrations with conventional lipidomics. This advantage of single cell analysis may enable for very early diagnosis of diseases like cancer or Alzheimer’s disease. Lipidomics analysis requires recording of the multitude of lipids present in cells, such as steroids, lipoproteins, phospholipids, sphingolipids, glycerides and fatty acids. Phospho- and sphingolipids constitute the essential biomolecules of cell membranes whilst glycolipids are the key molecules in cell recognition and also immunological processes. The high number of analysed compounds is resulting in the generation of huge data sets. Therefore lipidomics studies require significant statistical data interpretation involving multivariate data analysis like principal component or discriminant analysis.

### 6.1. Differentiation of Mammalian Cell Types

ToF-SIMS analysis in combination with multivariate statistical analysis was used to differentiate between mouse fibroblasts and rat oesophageal epithelial cells in heterogeneous cultures [47]. For this purpose a multivariate statistical model was established from pure cultures of both cell types. Using this model unknown regions of a mixed culture were analysed and back-feeded into the established statistic model. The approach could successfully separate the two different cell lines from each other and was further successfully applied for their identification in co-culture experiments.

### 6.2. Phospholipid Composition Influences on the Communication Capacity of Neighbouring Cells

Rat derived neuronal PC12 cells that communicate via exocytosis were shown to express a time-dependent uptake of stable isotope-labelled phospholipids. Biomolecules labelled with stable isotopes (^13^C, ^15^N or ^10^B) were added to the culture medium. Simultaneous incubation of PC12 cells with phosphatidylcholine resulted in a decrease of exocytosis, whilst the cultivation with phosphatidyl ethanolamines resulted in an increased exocytosis rate [48]. The incubation experiments showed on a single cell basis that the incorporation rate for labelled dipalmitoyl phosphatidylcholine and dipalmitoylphosphatidyl ethanolamine was 0.5% or 1.3% respectively [34]. This demonstrated for the first time that rather small differences in cell membrane phospholipid overall composition of single cells can result in significant changes in exocytosis rates in PC12 cells and therefore influence the cellular communication capacity with neighbouring cells.

### 6.3. Differentiation of Macrophages Exposed against Silver Nanoparticles

Human-derived macrophages exposed to silver nanoparticles could be successfully distinguished from the untreated controls by the application of ToF-SIMS. Size and surface modification of the nanoparticles were shown to influence the effect of N-acetyl cysteine pretreatment, which was applied to nanoparticle-exposed cells for a reduction of oxidative stress [49,50].

### 6.4. Phospholipid Pattern of Yeasts Enables for Species Discrimination

**Figure 1:**
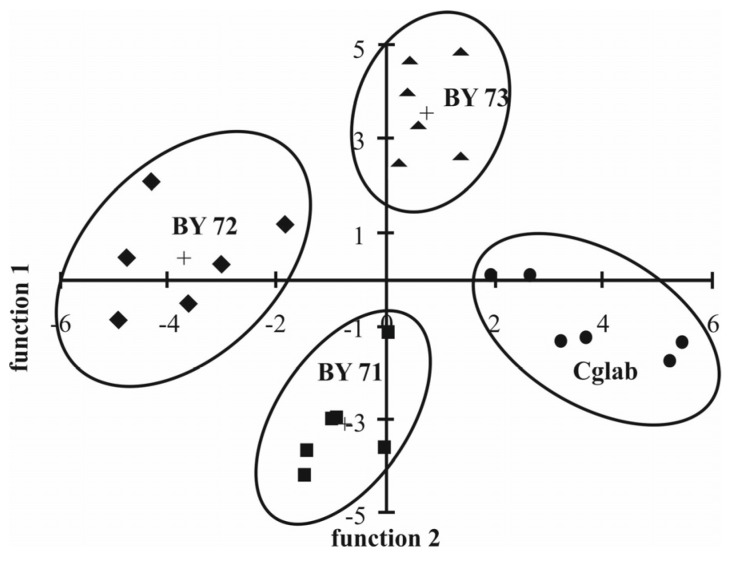
Values of two discriminant scores (discriminant analysis) for the 24 yeast samples for 34 principal ions, which were selected to discriminate between *S. cerevisiae* (haploid strains BY4741 and BY4742) (BY71 and BY72), *S. cerevisiae* diploid strain BY4743 (BY73), and *C. glabrata* (Cglab). *S. cerevisiae* (haploid strain BY4741) group; +. group centroids. Figure reprinted with permission from [51]. Copyright 2005, American Chemical Society.

**Figure 2:**
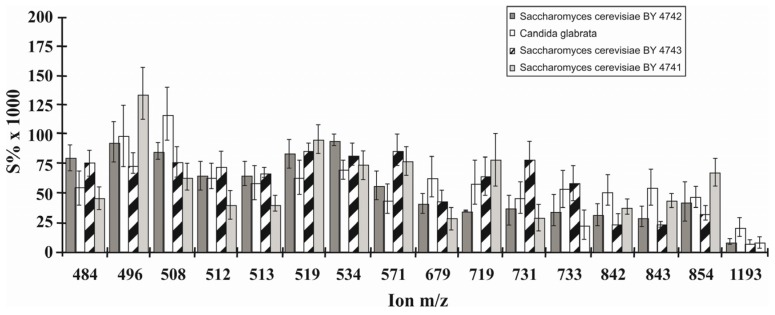
Relative abundances of characteristic ions (450–1200 Da) used to separate four different yeasts: *S. cerevisiae* (haploid strain BY2742), *C. galbrata*, *S. cerevisiae* (diploid strain BY4743), and *S. cerevisiae* (haploid strain BY4741). The *x*-axes shows the selected ions; the *y*-axes shows the relative ion abundance (calculation: S% × 1000). Figure reprinted with permission from [51]. Copyright 2005, American Chemical Society.

The cellular surfaces of four different yeast strains belonging to the species *Candida glabrata* and *Saccharomyces cerevisia* were evaluated by a multivariate statistical analysis of ToF-SIMS data [51]. A total of 24 samples could be assigned to one of the strains (Figure 1). Thirtyfour ions were found sutiable for differentiation of the strains by application of principal components analysis in combination with discriminant function analysis and cluster analysis. While 16 ions of molecular mass of 450 up to 1000 were assigned to phospholipid species (Figure 2), 18 ions were assumed fragments. As the approach was focussed on consistent differences between the strains a selection of uncommon compounds occuring in low concentrations was assumed. 

### 6.5. Identification of a Diatom Microalgae in a Microbial Mat

The diatom microalgae species *Planothidium lanceolatum* was identified in a complex marine microbial consortium of a microbial mat, 183 m below sea level, by the use of ToF-SIMS in combination with single cell lipidomics studies. Cellular lipid marker compounds like fatty acids, mono-, di- and triglycerides and carotenoids were successfully used to distinguish single diatom microalgae from other microbes of the consortium. Furthermore, this methodology has enabled the study of environmental influences on lipid metabolism of specific microorganisms in complex microbial consortia without prior sample preparation [52].

### 6.6. Lipid Formation Changes in the Single Cell Organism Tetrahymena Thermophila during the Mating Process

ToF-SIMS in combination with lipidomics was used to show that the lipid domain formation in the mating region of *Tetrahymena thermophila* can be identified by a local decrease in phosphatidylcholines [53]. This decrease can be observed in the mating junction region in a time-dependent manner. The same study could also show that the lipid domain formation correlated directly with pore formation and trituration resistance indicating a stronger cell pairing in mating cells containing the lipid domains. These results show that ToF-SIMS, due to its high chemical specificity in combination with submicron lateral resolution is very well suited to directly visualise lipid domains. 

### 6.7. Lipid Profiling of Xenopus Laevis Embryos

The lipid pattern of the *Xenopus laevis* egg membrane during fertilization and development of the two-cell embryo was visualized with ToF-SIMS using a 40 keV C_60_^+^ primary ion source. For evaluation of mass spectral data, a principal component analysis was conducted. Distinct regions on the zygote surface with chemical gradients, not present in the unfertilized egg, were identified and indicated as potential sperm entry points. Diacylglycerol and cholesterol were described as most abundant in the center of these sites at this time point. During the other stages of the egg development, cholesterol and diacylglycerol appear to distribute homogenous over the surface of the potential egg-sperm fusion sites, cholesterol potentially with a function in stabilisation of newly synthesised membrane. The study demonstrates the potential of ToF-SIMS to investigate the role of lipid distributions in the biological processes of single cell development [54].

### 6.8. Palmitoleic Acid Content of Human Breast Cancer Stem Cells is Reduced 

ToF-SIMS analysis employing a 60 keV gold cluster gun was performed on membrane lipids of human breast cancer stem cells, a population possessing the ability to regenerate tumours following to their regression due to therapy. Cells were isolated from human sample material using fluoresecence activated cell sorting (FACS). The ratio of intensity observed for palmitoleic acid, m/z 253.2, was determined with 43.2% between human breast cancer stem cells and non-stem cancer cells. Neither further differences in fatty acid composition nor specific membrane structures were observed. The study may give hints for cancer therapy and represents an example for the application of FACS and ToF-SIMS for imaging of cellular membrane components [55].

### 6.9. Potential Skin Penetration of Cosmetic Ingredients

Lipidomics linked to imaging mass spectrometry for single cell analysis is further representing a suitable tool for investigation of potential penetration mechanisms of cosmetic ingredients related to their fate and potential toxicity. ToF-SIMS analysis was used to localise commonly applied susbtances in the dermis and in the stratum corneum, representing the major part of the skin barrier consisting mainly of corneocytes and extracellular lipid matrix [56]. The study showed that oleic, lauric and capric acid, which are used as chemical penetration enhancers in the cosmetics industry, could readily penetrate the human skin. However, only oleic acid could enhance the overall uptake of tolnaftate, a lipophilic anti-fungal agent used as a model substance.

## 7. Conclusions

Molecular imaging offers a new perspective for the 3-dimensional analysis of cellular substructures on a microscale, envisioning the possibility not only to look at cellular compartments but also at the biomolecules associated with each compartment and its metabolic flux. With the advent of cluster ion guns and the possibility for 3-dimensional, mass spectrometry-based chemical imaging, especially ToF-SIMS and magnetic sector field SIMS are very well suited for that purpose. Three-dimensional reconstruction of subcellular structures like the nucleus or the mitochondria with full chemical distribution patterns including exact quantification will allow new insights in cellular uptake processes and in the regulation of signalling pathways. The identification of possible metabolic compartments may shed further light on regulation, transport and metabolism of chemicals and drugs. 

Especially for pharmaceuticals, the uptake rate and intracellular 3-dimensional distribution of the active ingredient are of paramount interest. Ideally the pharmaceutical acts not only organ specific and within the target organ, e.g., tumour cell specific, but can also target specific intracellular compartments for enhanced drug performance. By this a significant reduction of active ingredient and negative side effects, caused by non-target supply will be achievable. This might allow the further development of personalised medicine and drug treatment regimens. 

For environmental chemicals molecular imaging offers the opportunity to investigate barrier penetration and cellular uptake mechanisms. To date, such information is missing to a large extent, especially considering the multitude of potential impurities in consumer products such as mineral oils and polycyclic aromatic hydrocarbons.

Furthermore, early changes associated with toxic effects, not visible on tissue or organ level, may be revealed by analysis of 3-dimensional distribution patterns of externally added compounds and metabolites in single cells. Environmental chemicals are predominantly compounds that cannot be biosynthesised by the living organism itself and therefore require cell-specific detoxification before excretion can occur. Such mechanisms may be activated only after the substances enter a specific single cell or have reached a certain intracellular concentration. This threshold may differ between organ tissues and single cells, resulting in different activation of specific metabolic and signalling pathways. Therefore different cells from a population may show an individual response according to their chemical load, which impacts on signalling patterns as well as enzyme and metabolite configurations. With the still necessary improvements, in particular regarding spatial resolution and sensitivity on biological samples, mass spectral imaging has the potential to discover such communication networks in order to facilitate toxicological risk assessment of chemicals originating from environmental sources and consumer products. 
